# Mental health characteristics of re-entering dropout Indian students

**DOI:** 10.6026/9732063002001034

**Published:** 2024-09-30

**Authors:** Baskaran M

**Affiliations:** 1 Department of Mental Health Nursing, PSG college of Nursing, Coimbatore, Tamil Nadu - 641004,India; 2 The Tamil Nadu Dr. M.G.R. Medical University, Chennai, India

**Keywords:** dropout, re-entering students, mental health, educational settings

## Abstract

Education plays a pivotal role in shaping individual development and societal progress, yet dropout rates remain a significant challenge, particularly in developing countries like India. Dropout decisions often stem from complex social, economic and psychological factors, impacting students' mental health. This comparative descriptive study assessed and compared the mental health characteristics of 30 re-entering dropout students and 30 regular students from Integral Coach Factory (ICF) Hr. Sec. School, Ayanavaram, Chennai. Data collection utilized a structured questionnaire capturing demographic variables and the modified Abraham and Prasanna Mental Health Characteristics Scale. Re-entering dropout students exhibited lower mental health scores across physical, intellectual, familial, social, and psychological dimensions compared to regular students (p < 0.001). Significant associations were observed between mental health and father's education level among re-entering dropouts (p < 0.001), emphasizing its influence on well-being The study underscores disparities in mental health between re-entering dropout students and their peers, highlighting the need for targeted interventions and supportive environments in educational settings. Addressing these disparities can enhance overall well-being and academic success among re-entering dropout students.

## Background:

Education is fundamental to individual development and societal progress, shaping the future of young generations worldwide. However, the journey through education is not always smooth, particularly for a significant proportion of students who experience disruptions such as dropping out of school [1]. The phenomenon of dropout rates among students, especially in the context of developing countries like India, poses substantial challenges to educational systems and societal well-being. Globally, dropout rates among school-aged children present a pressing issue [2]. In India a study cross-sectional community-based study in Raipur, Chhattisgarh, found 11% scholastic dropouts among adolescents. While, poor academic performance is another determining factor [3]. These figures underscore a critical need to delve deeper into the factors contributing to school dropout and the subsequent implications for mental health. The decision to drop out of school often reflects underlying social, economic, and psychological challenges faced by students and their families [4]. Studies have consistently linked school dropout to increased risks of mental health issues such as emotional disturbances, behavioural disorders (*e.g.*, conduct disorder, attention deficit hyperactivity disorder), and overall compromised well-being [5-6]. Therefore, it is of interest to report the mental health dynamics of re-entering dropout students is crucial for fostering inclusive and supportive educational environments.

## Methodology:

## Study design:

This study employed a comparative descriptive design [7] to assess and compare the mental health characteristics of re-entering dropout students and regular students in UDAVI, Chennai, India.

## Participants:

The study employed purposive sampling to select 30 re-entering dropout students and 30 regular students from Integral Coach Factory (ICF) Hr. Sec. School, Ayanavaram, Chennai, TN, India. The study included 30 re-entering dropout students and 30 regular students who met the following inclusive criteria:

[1] Ability to read and write in Tamil and English.

[2] Attendance at Integral Coach Factory (ICF) Hr. Sec. School, Ayanavaram, Chennai.

Exclusion criteria involved re-entering dropout students with diagnosed psychiatric illnesses, ensuring that participants did not have pre-existing conditions that could confound the study outcomes.

## Setting:

Data collection took place at Integral Coach Factory (ICF) Hr. Sec. School, Ayanavaram, Chennai, providing a suitable environment familiar to both groups of participants.

## Instrumentation:

## Section A:

Demographic variables

A structured questionnaire was used to gather demographic information

## Section B:

Modified Abraham and Prasanna mental health characteristics scale

The primary instrument used to assess mental health characteristics was a modified version of the Abraham and Prasanna Mental Health Characteristics Scale (1978), developed at the Department of Education, University of Kerala. This scale, originally comprising 80 items across 16 sections.

## Data collection procedure:

Data collection occurred over a specified period, during which trained researchers administered the questionnaire and mental health scale to participants. The process ensured consistency and reliability in data collection across both groups.

## Data analysis:

Both descriptive and inferential statistical analyses were employed to analyze the data.

## Ethical considerations:

The study adhered to ethical guidelines, ensuring voluntary participation, confidentiality, and anonymity of participants. Informed consent was obtained from all participants or their legal guardians, and ethical approval was obtained from the relevant institutional review board.

## Results:

Table 1 presents the demographic characteristics of the participants, showing notable differences between the two groups. It shows that re-entering dropout students are more likely to belong to families with lower educational backgrounds and lower monthly incomes compared to regular students. For instance, 46.7% of re-entering dropout students' fathers and 43.3% of their mothers were illiterate, compared to only 23.3% and 26.7%, respectively, in the regular student group. Additionally, most re-entering dropout students (76.7%) came from families with incomes between Rs. 1001-3000 per month, whereas a higher proportion of regular students (40%) belonged to families earning above Rs. 3000 per month. These demographic differences suggest that socioeconomic factors may play a critical role in influencing dropout behavior and subsequent mental health outcomes.

Table 2presents the mean and standard deviation (S.D.) scores for mental health dimensions in both groups, along with the corresponding t-values and significance levels. Re-entering dropout students had lower mean scores across all dimensions-physical (12.97 vs. 10.23), intellectual (12.13 vs. 9.37), familial (11.77 vs. 9.97), social (12.97 vs. 10.37), and psychological (12.3 vs. 9.87)-compared to regular students. The t-values indicate highly significant differences (p < 0.001) for each dimension, emphasizing the poorer mental health status of re-entering students. These results underscore the need for targeted psychological and social interventions to address the challenges faced by this group.

**Figure 1:**
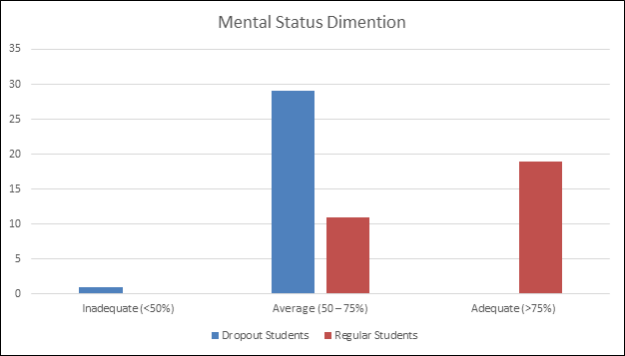
Comparison of Mental status dimension among re-entering dropout students and regular students

Figure 1 compares the mental health characteristics of dropout and regular students, showing that 29 dropout students fall in the average range (50-75%), with none achieving an adequate level (>75%), unlike 19 regular students who scored adequately. Only 1 dropout student displayed inadequate mental health (<50%), while no regular students fell in this category. These results highlight significant mental health disparities, emphasizing the need for targeted support for dropout students to enhance their well-being.

## Discussion:

The present study aimed to explore and compare the mental health characteristics of re-entering dropout students and regular students in UDAVI, Chennai, India. In our study, re-entering dropout students predominantly exhibited an average level of mental health across various dimensions-physical, intellectual, familial, social, and psychological. This finding aligns with the study conducted by Wainipitapong *et al.* (2022), which also reported similar levels of poor to average mental health among re-entering students [8]. However, in contrast to our findings, the study by Hjorth CF *et al.* (2016) found a higher prevalence of inadequate mental health levels among re-entering students, suggesting varying degrees of mental health challenges upon returning to education [9]. In the present study, the data indicate that the majority of regular students exhibit adequate to good mental health, consistent with findings from studies conducted by Siddique *et al.* (2022) and Pedrelli *et al.* (2015) [10, 11] Our study highlighted significant differences in mental health levels between re-entering dropout students and their regular counterparts. Re-entering dropout students consistently showed lower mental health scores across all measured dimensions compared to regular students. This pattern is consistent with findings from the study by Del Savio *et al.* (Year), which also noted disparities in mental health between dropout students re-entering education and their peers who remained in school without interruption. These consistent findings underscore the robustness of the impact of dropout experiences on subsequent mental health outcomes [12]. The association analysis in our study revealed a significant link between father's education level and mental health outcomes among re-entering dropout students. This finding aligns with the research conducted by Fakhrunnisak *et al.* (2022), which similarly identified parental education as a significant factor influencing mental health among adolescents and young adults [13]. However, unlike Varsha V et al (2023) and Lindhardt *et al.*'s (2022) findings, we did not observe significant associations between mental health and demographic variables such as age, sex, class, and religion among re-entering dropout students. These contrasting results suggest potential contextual variations that warrant further investigation in future studies [14, 15]. Despite the contributions of our study, limitations such as sample size and geographical specificity should be considered. Future research could expand on these findings by incorporating larger and more diverse samples, as well as exploring additional demographic and contextual factors that may influence mental health outcomes among re-entering dropout students. In conclusion, our study contributes valuable insights into the mental health characteristics of re-entering dropout students compared to regular students. By drawing comparisons with findings from multiple studies, we underscore the need for tailored interventions and support mechanisms to address mental health disparities and enhance overall well-being among re-entering dropout students in educational settings.

## Figures and Tables

**Table 1:** Characteristics of Re-entering Dropout Students and Regular Students

**Demographic Variables**	**Re-entering Dropout Students (n=30)**	**Regular Students (n=30)**
Age		
10-12 years	12 (40.0%)	-
13-15 years	16 (53.3%)	22 (73.3%)
Above 16 years	2 (6.7%)	8 (26.7%)
Sex		
Male	15 (50.0%)	15 (50.0%)
Female	15 (50.0%)	15 (50.0%)
Class		
5th standard	10 (33.3%)	9 (30.0%)
6th standard	7 (23.3%)	8 (26.7%)
7th standard	2 (6.7%)	8 (26.7%)
8th standard	11 (36.7%)	5 (16.7%)
Religion		
Hindu	14 (46.7%)	7 (23.3%)
Christian	9 (30.0%)	14 (46.7%)
Muslim	7 (23.3%)	9 (30.0%)
Others	-	-
Father's Education		
Illiterate	14 (46.7%)	7 (23.3%)
Primary	9 (30.0%)	14 (46.7%)
Secondary	7 (23.3%)	9 (30.0%)
Mother's Education		
Illiterate	13 (43.3%)	8 (26.7%)
Primary	10 (33.3%)	19 (63.3%)
Secondary	7 (23.3%)	3 (10.0%)
Type of Family		
Nuclear	21 (70.0%)	21 (70.0%)
Joint Family	9 (30.0%)	9 (30.0%)
Living Status of Parents		
Staying Together	25 (83.3%)	29 (96.7%)
Separated	5 (16.7%)	-
Widow/Widower	-	1 (3.3%)
Caretaker (or) Guardian		
Parents	29 (96.7%)	27 (90.0%)
Hostel Warden	-	2 (6.7%)
Relatives	1 (3.3%)	1 (3.3%)
Family Income (Rs/month)		
Rs. 500-1000	1 (3.3%)	-
Rs. 1001-3000	23 (76.7%)	18 (60.0%)
Rs. 3001-5000	4 (13.3%)	7 (23.3%)
Rs. 5000 above	2 (6.7%)	5 (16.7%)
Number of Siblings		
One	1 (3.3%)	9 (30.0%)
Two	23 (76.7%)	11 (36.7%)
Three	4 (13.3%)	6 (20.0%)
Four and above	2 (6.7%)	2 (6.7%)
Nil	-	1 (3.3%)
Order of Birth		
First	7 (23.3%)	13 (43.3%)
Second	12 (40.0%)	8 (26.7%)
Third	4 (13.3%)	6 (20.0%)
Fourth	7 (23.3%)	3 (10.0%)

**Table 2:** Comparison of mean and S.D of mental health between the re-entering dropout students and regular students

***Dimensions***	**Re-entering dropout students**		**Regular students**		**t'value**
	**Mean**	**S.D**	**Mean**	**S.D**	
1.Physical	12.97	1.87	10.23	1.1	6.90*** (S)
2.Intellectual	12.13	2.03	9.37	2.01	5.30*** (S)
3.Familial	11.77	1.25	9.97	1.13	5.85*** (S)
4.Social	12.97	2.25	10.37	0.89	5.89*** (S)
5.Psychological	12.3	2.18	9.87	1.46	5.07*** (S)
Overall	49.8	3.6	62.13	6.45	9.133***(S)
